# Supplementary material for: Learning curve and surgical outcome of robotic assisted colorectal surgery with ERAS program
Source: Sci Rep. 2022 Nov 29;12:20566. doi: 10.1038/s41598-022-24665-w (PMC9709162; doi:10.1038/s41598-022-24665-w)
Supplement: Supplementary file 2 — Supplementary Information 2. [file 41598_2022_24665_MOESM2_ESM.zip › RAL Raw data-Ñ[▒K/116/OP N_ñw╝╨░O▒Kñσ.pdf]

W92 / W92

臺 中 榮 民 總 醫 院

手術室護理記錄

[redacted]

基本資料

- 室別： 開刀房-17室，手術科別： CRS，手術類別： 常規，日期： 2021/10/07
- 進入時間： 1425 ； 麻醉開始時間： 1430 ； Time Out時間： 1553 ； 劃刀時間： 1554 ； 手術結束時間： 2035 送出時間： 2100 ；
- 麻醉方式： GE
  診斷： Malignant tumor of cecum
  手術名稱： Robot Assisted Colectomy(R)
  手術局部用藥： nil
  術前預防性抗生素：
  - 抗生素名稱一： Veterin 1GM IVD(由麻醉科給予)
  - 時間： 1515
  - 上次簽章: 劉雅汶

  ，追加預防性抗生素：
  - 抗生素名稱一： Veterin 1GM IVD(由麻醉科給予)
  - 時間： 1915
  - 上次簽章: 劉雅汶
- 手術醫師： 蔣鋒帆，陳瑨夫，陳凱祥
- 麻醉醫師： VS 陳永銘
-
  - 刷手護士A ： 歐真勤，時間： 1425，刷手護士B ： 黃彥翔，時間： 1425，接替刷手一： 許芷瑄/蘇郁安，時間： 1500
  - 流動護士A ： 劉雅汶，時間： 1425，接替流動一： 蘇郁安，時間： 2000

皮膚狀況

A-V Shunt： 無
術前： 完整
  記錄者： 上次簽章: 劉雅汶
術後： 完整
  記錄者： 蘇郁安

手術準備

手術臥式 單一手術臥式

-
  - 手術臥式： 仰臥
  - 使用附件： 溫毯 38 ℃，頭圈
  - 皮膚準備： 10%Aq Betadine，Hibiscrub，2% Chlorhexidine
  - 電燒： 使用電燒: 電極片位置： 其它： 電燒回路軟墊，雙極電燒，記錄者： 上次簽章: 劉雅汶

止血帶T： 無

植入物

- 植入物名稱/號碼/數量/部位： Covidien GIA 80mm-3.8mm*2/cecum

，記錄者： 上次簽章: 劉雅汶

標本送檢

- 細菌培養 無
- 細胞學 無
- 冰凍切片 無
- 病理標本
  組織名稱一： A colon
- 截肢 無

標本交付 標本： 1 袋，標本： 0 盒，標本： 0 瓶，細胞學： 0 管，Culture Tube： 0 管，被交付醫師： CR 陳瑨

夫，

- 記錄者： 上次簽章: 劉雅汶
- 時間： 1900

傷口術後敷料

傷口敷料：　　　　傷口敷料： 無

- 請於圖上標示部位：

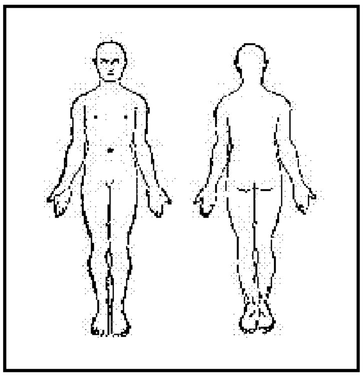

- 傷口敷料： trocar傷口:軟性敷料

導管

導管	天數	導管資訊	插入日期	插入時間	更換日期	拔除日期	拔除時間	拔除原因	預計拔除/更換	預計拔除/更換日期
A-line (1)	1	號碼:20 Ga; 部位:右手;	2021/10/07	1525						
Foley(1)	1	號碼:16 Fr.; 部位:膀胱;	2021/10/07	1530						
Double Lumen CVC(1)	1	號碼:7 FR; 部位:jugular (右); 象限:RU;	2021/10/07	1500						
Epidural PCA(1)	1	部位:硬脊膜下;	2021/10/07	1300						

用物計數

- 手術中出血量： minimal c.c.
- 手術中尿量： 550 c.c.
-
    - 其它： 無

用物計數： 多切口同時縫合

- 1.器械： 正確，2.縫針： 正確，3.刀片： 正確，4.紗布： 正確，5.腹部墊： 未使用，6.紗球： 未使用，7.PEANUTS ： 未使用，8.紗條： 未使用，9.絲帶： 未使用，10.針頭： 未使用，11.Vessel loop： 未使用，12.Boots： 未使用，13.Jelco： 未使用，14.棉條： 未使用，
    - 第一次：
        - 刷手護士 ： 許芷瑄
        - 流動護士： 上次簽章: 劉雅汶
        - 時間： 1915

  ,
    - 第二次：
        - 刷手護士 ： 許芷瑄
        - 流動護士： 上次簽章: 劉雅汶
        - 時間： 1930

  ,
    - 第三次：
        - 刷手護士 ： 許芷瑄
        - 流動護士： 上次簽章: 劉雅汶
        - 時間： 1945

備註

備註： davinci 30度 scope

達文西上機時間:1635
達文西下機時間:1806

- 記錄者： 上次簽章: 劉雅汶
- 時間： 1550

填寫護理人員_蘇郁安

正式摘要

W92 / W92

doctorRank:B
